# Impact of Age on the Prognosis of Operable Gastric Cancer Patients: An Analysis Based on SEER Database: Erratum

**DOI:** 10.1097/01.md.0000494763.03121.ed

**Published:** 2016-08-26

In the article “Impact of Age on the Prognosis of Operable Gastric Cancer Patients: An Analysis Based on SEER Database”,^1^ which appeared in Volume 95, Issue 24 of *Medicine*, Dr. Jie Chen's surname was omitted in the byline. The article has since been corrected online.
